# Food Safety for First Responders

**DOI:** 10.3201/eid1103.040700

**Published:** 2005-03

**Authors:** Mary K. Afton, Michele Nakata, Myra Ching-Lee, Paul V. Effler

**Affiliations:** * Hawaii Department of Health, Honolulu, Hawaii

**To the Editor:** Relatively few published reports of intentional food contamination are available (1–5). However, after the anthrax attacks of 2001, biologic terrorism vulnerability assessments have determined that intentional food poisoning is a plausible means to widely disseminate pathogens, with potentially devastating effect (6). As a consequence, food security has emerged as one of the major priorities for bioterrorism preparedness (7–10). We describe a naturally occurring incident that demonstrates the potential for premeditated food contamination to target specific populations who are critical for protecting public safety, in this instance, a city police force. Measures to mitigate the risk of this scenario type are provided.

On the evening of December 19, 1998, local hospital emergency departments notified the Hawaii Department of Health that an unusually high number of police officers were coming to the hospitals with acute gastroenteritis. Earlier that day, several police-affiliated support associations had cosponsored a holiday event. The food was catered by a food service establishment that routinely provided meals at the police department headquarters in Honolulu. Active and retired police officers and their family members participated in the event. The food service at the event consisted of "bento lunch boxes" containing luncheon meat, a hot dog, teriyaki beef, fried chicken, and rice. Approximately 1,100 lunches were distributed at the event, some of which were taken offsite to be consumed by others, including on-duty officers at satellite police stations.

Interviews were conducted with a convenience sample of 394 persons who ate the bento lunch, of which 145 (37%) reported becoming ill with diarrhea (81%) or vomiting (80%). Illness onset occurred a mean of 4 hours after eating the lunch (1.5–8 hours); the mean duration of illness was 14 hours (2–96 hours). Incapacitation of police officers and their family members as a result of the illness was substantial with 25% absent from work for a half day, 42% for 1 full day, 18% for 2 days, and 15% for more than 2 days. *Staphylococcus aureus* was recovered from 7 of 8 stool specimens, of which 6 were positive on *S. aureus* toxin testing. Analyses of the lunch items found between 18 million to 3 billion *S. aureus* colonies per gram in the implicated foods. Luncheon meat, teriyaki beef, and hot dogs were positive on *S. aureus* toxin assays. *S. aureus* isolates obtained from food and clinical specimens were indistinguishable by pulsed field-gel electrophoresis. The quantity of lunch boxes produced for this holiday event exceeded that which the catering facility routinely produced while adhering to recommended food-handling guidelines. A facility inspection and review of the caterer's procedures identified improper holding temperatures for potentially hazardous foods as the likely cause of the outbreak.

In this incident, prompt action by the police department, which employed an agency-wide radio communications system to warn officers not to eat lunches obtained for later consumption, reduced the number of persons who would have become ill from staphylococcal foodborne infection. In spite of this effort, the outbreak still had a considerable impact on the staff of the police department. The attack rate for those exposed was high, and three quarters of those who became ill missed >1 days of work. If an agent causing greater severity of illness, e.g., botulinum toxin, had been introduced into the bento lunches instead of *S. aureus*, the ensuing outbreak might have strongly compromised the department's ability to ensure public safety.

Despite modifications in food security industry regulations because of the Bioterrorism Act of 2002, intentional contamination of food items on a smaller scale remains a potential danger that needs to be addressed. As the incident we described demonstrates, under certain circumstances, terrorists may be able to substantially impair first response agencies, including police departments, through a limited but targeted foodborne attack. By incapacitating first responders, terrorists might maximize the impact of a larger, coordinated event. Just as maintaining the physical security of the Strategic National Stockpile is a priority in preventing a secondary attack, reasonable steps must be taken to ensure that our emergency workforce is protected from a targeted foodborne assault.

As a result of this outbreak, we have recommended that whenever entire units, departments, or shifts of first responders in our jurisdiction are involved in shared dining activities, efforts should be made to obtain food items from more than 1 caterer for each meal. If departmentwide events necessitate using a single caterer, efforts should be taken to identify and mitigate the threat of intentional food tampering and there should be rigorous adherence to standard safe food-handling procedures to minimize the potential for naturally occurring outbreaks (7). Our recommendations here are similar to those employed by airlines to protect pilots and copilots on long flights by serving separate meals prepared in different kitchens (11).

Our intention is to share with other preparedness agencies our observation that first response assets might be compromised by something as seemingly innocuous as a holiday party. Appropriate planning may reduce the risk of intentional food contamination that targets security forces or first responders, either as an isolated strike or as part of a larger, coordinated terrorist attack.
